# Patient's Adherence and Compliance and Quality of Life During/After VIT

**DOI:** 10.3389/falgy.2022.886054

**Published:** 2022-06-28

**Authors:** Cristoforo Incorvaia, Enrico Heffler, Silvia Peveri, Francesco Pucciarini, Giorgio Walter Canonica, Erminia Ridolo

**Affiliations:** ^1^Senior Consultant, Post-graduate School of Allergology and Immunology, University of Parma, Parma, Italy; ^2^Department of Biomedical Sciences, Humanitas University, Pieve Emanuele, Italy; ^3^Personalized Medicine, Asthma, and Allergy, IRCCS Humanitas Research Hospital, Rozzano, Italy; ^4^Allergy Department Unit, Piacenza Hospital, Piacenza, Italy

**Keywords:** VIT, quality of life, adherence–compliance–persistence, fire ant allergy, fire ant immunotherapy, mastocytosis, vespid allergy, hymenoptera allergy

## Abstract

Adherence and compliance, respectively considered as a more positive, proactive behavior, resulting in a patient's lifestyle change to follow a daily regimen, and, as a more enforced response to an external command, are a critical aspect of any medical therapy, since it is estimated that less than half of the patients who are prescribed a therapy perform it, respecting the doses and duration. As far as aeroallergen immunotherapy is concerned, current data show that adherence is respected in about 50% of subcutaneous immunotherapy and in percentages even lower than 20% in sublingual immunotherapy treatments. This review analyzes the adherence to venom immunotherapy (VIT), in which, given its purpose of preventing potentially fatal anaphylactic reactions to insect stings, this aspect plays a critical role. In fact, protection from stings already takes place when the maintenance dose is reached, but VIT interruption before the recommended duration of 5 years exposes patients to new sting reactions. The data on adherence to VIT are far less abundant than that for aeroallergen immunotherapy. One of the first studies reported poor adherence in Austria, but the model used, consisting in the estimate of the percentage of patients with systemic reactions who accepted or rejected VIT, does not meet the criteria that define adherence to treatment. As for appropriate adherence studies, rates higher than 70% were reported in the United States and European countries. Studies from Italy found that good adherence were observed also in patients receiving, after 4 years of VIT, 3 months extended maintenance dose, as well as in patients treated during the COVID-19 pandemic, <10% of whom stopped VIT. Instead, only 35% of the patients treated for allergy to imported fire ant remained adherent after 1 year of treatment. However, also concerning honeybees and vespids, although adherence is satisfactory, it is possible to further improve it by increasing information and support for patients. Health-related quality of life (HRQL) is an efficient measure to estimate the effectiveness and safety of medical treatment. Tools designed to make patients aware of its improvement through VIT and, in particular, of the complete prevention of the risk of fatal reactions have an important role in reinforcing adherence. However, aspects not yet evaluated, such as the possible relationship between the efficacy of VIT and HRQL or its particular features in patients with mastocytosis, deserve specific studies.

## Introduction

This review analyzes adherence to venom immunotherapy (VIT), in which the risk of severe reactions to insect stings if the treatment is performed irregularly makes adherence of critical importance. In general, this can be verified in any treatment by measuring the degree to which the therapy is followed through the evaluation of the patient's adherence and compliance. According to a recent appraisal, adherence is considered more participatory and related to the individual's belief that something is of value to him, while compliance is more enforced, as a response to an external command (1). Actually, there is broad evidence that the patients' regularity in following the prescriptions is insufficient and is coming to represent a major medical problem. A valuable analysis from Cutler and Everett in 2010 showed that overall non-adherence affects more than 50% of patients who are prescribed drug therapy (2). However, the rate of adherence can be very uneven according to the different therapies in analysis. This is particularly relevant in regard to allergen immunotherapy (AIT), whose action is not limited to the symptoms' control but has a disease-modifying effect; this benefit of AIT, however, takes place only if the treatment is continued regularly for at least 3 consecutive years (3). In an actual comprehensive review, Demoly et al. analyzed the issues related to lack of adherence and compliance (4). First, a classification was used to define adherence in different stages, consisting of *initiation* (i.e., acceptance), *implementation* (i.e., compliance, as the proportion of the recommended doses of actually taken by the patient), and *persistence* (i.e., continuing the treatment to the end of the recommended period).

Globally, the compliance part of adherence has been estimated to be between 25% and 90% (5). The most discouraging aspect concerns sublingual immunotherapy (SLIT): the data from two major manufacturers of AIT products in Italy have shown that more than 50% of patients stopped the treatment during the 1st year, and only 13% continued it in the 2nd year (6). A similar result was reported in the Netherlands, where only 7% of SLIT treated patients reached the required duration of treatment of 3 years (7). These poor results have stimulated the search for interventions to improve adherence to AIT. They consist of accurate patient education at the beginning of treatment, sharing with patients the choice of the type and route of AIT, and showing real interest on their treatment. Also, an organized specialist time schedule, resulting in close follow-up and increase in patient loyalty, could successfully improve adherence (8).

Regarding VIT, since its most important role is the prevention of fatal reactions to stings, the importance of evaluating adherence to treatment is of particular interest.

## Adherence to Venom Immunotherapy

In patients with a history of systemic reactions to Hymenoptera stings, VIT already induces tolerance when the maintenance dose is reached (9). However, VIT's termination before the recommended duration of 5 years exposes patients to new sting reactions. Studies conducted on adherence to VIT are much less numerous than that the ones on AIT, probably because doctors believe that patients are aware of the life-saving effect of the therapy treatment and the need of long treatment duration. One of the first studies on the matter was performed in Austria, using the stage “initiation” cited above, reporting poor adherence to VIT. However, the simple estimate of the percentage of patients with systemic reactions to Hymenoptera stings in VIT-treated and untreated patients, respectively, cannot state adherence as defined by the available studies. Concerning appropriate adherence evaluation, the first study addressed the outcome in patients with severe systemic sting reactions, receiving, after 4 years of VIT, 3-month-extended maintenance treatment, reducing the number of doses to be administered. The patients were informed of the risk of relapse after early discontinuation. During the 3-month interval maintenance phase, the safety remained excellent, with only local reactions in 17.8% of the patients, and all equally good adherence was observed (10). Bilò et al. performed two studies on the issue. In the first, following a thorough per-patient counseling about efficacy, safety, and the need of a 5-year course to achieve long- lasting outcomes, 508 patients underwent VIT from January 2000 to December 2006, which resulted in adherence of 84% (11). The other study evaluated adherence, together with safety, of VIT during the COVID-19 pandemic. All the patients delaying VIT because of the pandemic were included in the study. The time extension was chosen based on the patient's risk profile (such as long pre-pandemic time interval, history of severe reaction, old age, and multiple comorbidities). The mean delay from the pre- to post-pandemic was 7 weeks and 15.5 weeks, respectively, while the total amount of the pre-pandemic VIT maintenance dose was safely administered in 78% of the patients. Thee of 87 patients had side effects; bee venom allergy and recent VIT initiation being identified as potential risk factors. The calculation of the adherence resulted in a value of 90.4%, showing a lack of influence of the complications imposed by the COVID-19 on VIT management (12). Indeed, the finding of higher adherence than the average values reported in the available studies suggests that the increased attention of physicians during the pandemic may have positively influenced the response of the patients. However, this hypothesis needs to be evaluated and eventually conformed in controlled studies.

Two revisions assessed the correlation between the adherence to VIT and the outcome of subsequent stings after discontinuation of treatment. Ilbilge et al. studied 55 children with moderate to severe systemic reactions to venoms for an overall period of 17 years, therefore well beyond treatment stopping. Long-term prognosis for re-sting reactions was good, some of the causes being refusing or discontinuing VIT being, possibly related to quality-of-life issues. Instead, preparedness of children on refusing or discontinuing VIT is low, especially concerning the correct use of adrenalin auto-injector in case of systemic reaction to re-sting. This observation suggested to authors the need of a multidisciplinary approach to increase the knowledge of pediatric patients (13). Also, Fiedler et al. performed a study on VIT-treated children surveyed for a mean period of 13 years, including 3 years of treatment and 10 years of follow-up. The outcome was less acceptable: anaphylactic relapses being not uncommon, with a considerable scarcity in emergency management of patients. Hence, the authors suggested to standardize anaphylaxis education programs as well as to schedule regular follow-ups of the allergy status in order to improve patient awareness and, consequently, adherence to treatments (14).

As has long been known, ants can also cause severe and potentially fatal allergic reactions. These insects are present in most temperate regions, but treatment with immunotherapy mainly concerns the United States, where the species most responsible for severe reactions requiring therapy is Solenopsis Invicta (Imported fire ant). Immunotherapy was prescribed since the 1970s with wide variability of outcomes. A recent review surveyed the outcomes of immunotherapy over 15 years compared to a similar 2009 study, finding a significant improvement in the accuracy and precision of immunotherapy dosing, the predominant dose being.5 ml 1:100 wt/vol, i.e., higher than those previously used (15).

So far, only one study has analyzed the adherence to imported fire ant immunotherapy, which attained the disappointing result that, despite the therapy having a potentially life-saving purpose, only 71% followed the starting recommendation, and, after 1 year, only 35% of the patients were still adherent. No statistically related factors, including age, gender, and severity of the initial allergic reaction, were individuated, while logistical constraints and fear were significant impediments. The authors concluded that physicians should be aware of the potential poor adherence to imported fire ant immunotherapy and be critical to consider in future studies logistical challenges and to maximize patient education concerning the risks-benefits ratio to improve adherence (16). Surprisingly, to date, this invitation has not been accepted, and, therefore, we do not know, other than VIT for apidae and vespidae, the degree of adherence to this type of immunotherapy.

## Quality of Life

Health-related quality (HRQL) of life is an efficient measure to estimate the efficacy and safety of any medical treatment (17). It is recognized that severe reactions to Hymenoptera stings are associated with a poor HRQL in adults and children (18, 19). The first studies on the ability of VIT to improve HRQL were performed in the Netherlands by Oude Elberink and co-workers. The initial study was limited to yellow jacket (Vespula species) therapy (20), the second included apidae and Vespidae (21), and the third found that VIT improves HRQL also in patients with skin reactions only, while those not choosing AIT and thus receiving a prescription of epinephrine auto-injector had a deterioration in HRQL (22). Cichocka-Jarosz et al. aimed at assessing the validity and reliability of adaptations of a common HRQL questionnaire to Polish vespid venom allergic children and their parents. The results showed that both adapted scales were valid and reliable tools to measure HRQL in children and their parents (23). Also, the Spanish version of an HRQH questionnaire for Hymenoptera venom allergy was validated, indicating that the awareness of tolerance to sting reduces the apprehension associated with the fear to have severe reactions when stung (24). The demonstration of acquired tolerance to Hymenoptera venom can also be obtained by performing a challenge by a sting of the responsible insect under medical supervision. Three studies are available, starting with that of Koschel et al., who performed a prospective investigation on 100 adult patients allergic to yellow jacket or honeybees, who filled in an HRQL questionnaire before and after a sting challenge. A statistically significant improvement was detected, which was independent of the patients' gender, age, and severity of the initial anaphylactic reaction. The authors concluded that the sting challenge results in a significant improvement in disease-specific HRQL (25). Fisher et al. found that a tolerated wasp sting challenge in VIT-treated patients improved HRQL (26). Such an outcome has been confirmed in a recent study, proving that the improved HRQL associated with VIT can be further increased by a tolerated sting challenge, that it is conceivable it can reassure the patients. A more marked effect was seen in the female gender (27). A fairly recent review has highlighted all aspects of Hymenoptera venom allergy and VIT, also considering adherence and quality of life, which were usually superior to those of other models of immunotherapy (28), although it remains to be clarified how much the results may vary according to the different centers that practice VIT (11).

## Conclusions

**Figure 1:**
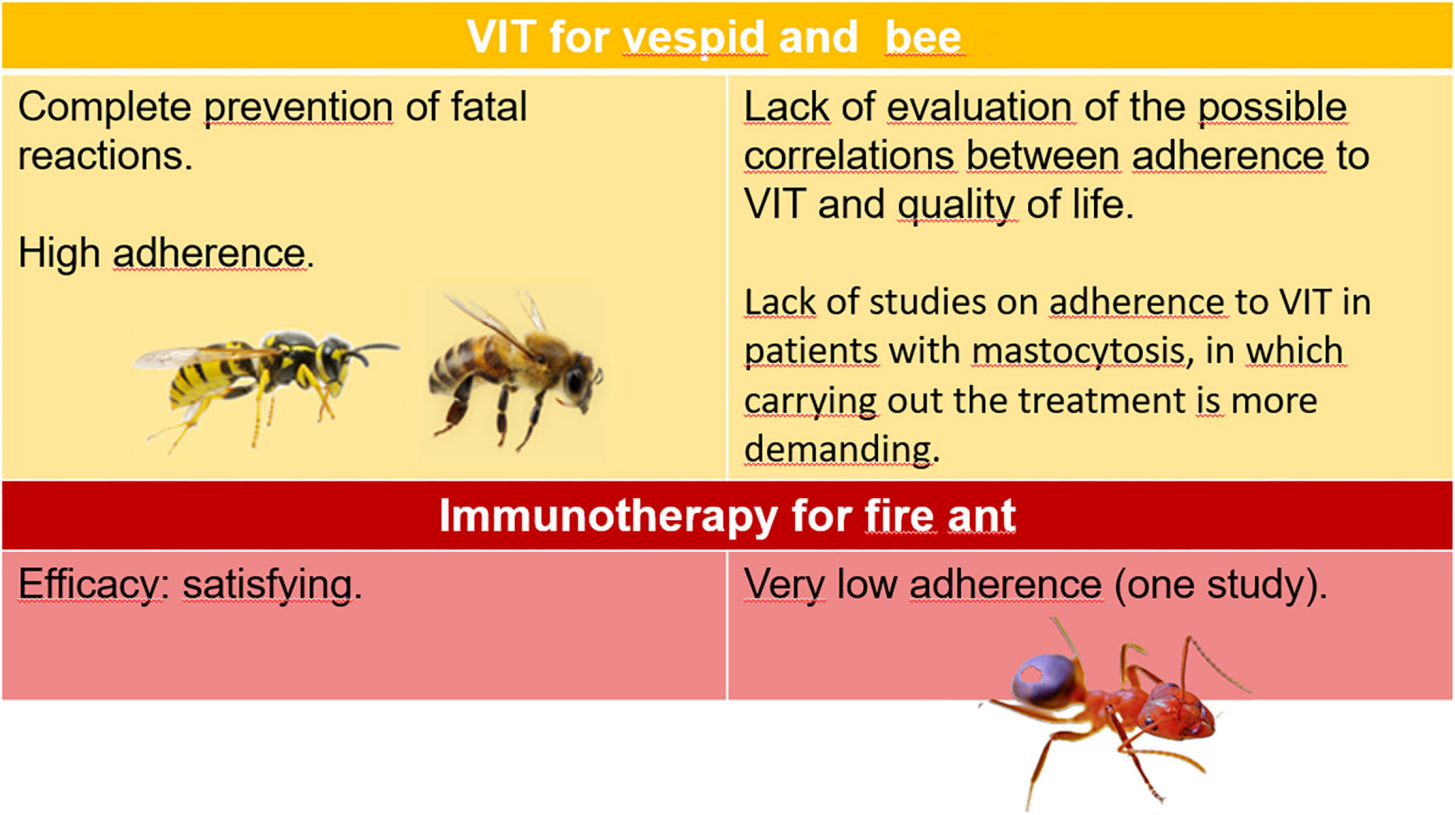
Certainties and shortcomings on adherence to VIT.

There is ample evidence on the reliability of diagnostic procedures and the effectiveness of VIT and sufficient knowledge of adherence to VIT with Hymenoptera venom from vespids and bees, while some aspects have not yet been clarified. One concerns adherence to imported fire antimmunotherapy; despite further studies to be compared to the only one available have been requested (15), the available data are insufficient to reach the level of evidence. Regardless of the different responsible Hymenoptera, the possible correlation between the effectiveness of VIT and its adherence has not been investigated so far. Another need to meet is to establish the degree of adherence to VIT in patients with mastocytosis, which has been, so far, unexplored, despite the particular needs of these patients, such as doubled maintenance dose and indefinite duration of the treatment (29). Figure 1 summarizes the state of knowledge of the different aspects of Hymenoptera venom allergy.

## Author Contributions

CI provided the idea, wrote the first draft, envisioned the picture, and contributed to the final revision. EH and SP helped with the first revisions and cured the bibliography. FP revised the manuscript and helped with the picture. GC helped in the elaboration of the first draft and the first revisions. ER provided the idea, wrote the first draft, contributed to the final revision, and managed the submission duties. All authors contributed to the article and approved the submitted version.

## Conflict of Interest

The authors declare that the research was conducted in the absence of any commercial or financial relationships that could be construed as a potential conflict of interest.

## Publisher's Note

All claims expressed in this article are solely those of the authors and do not necessarily represent those of their affiliated organizations, or those of the publisher, the editors and the reviewers. Any product that may be evaluated in this article, or claim that may be made by its manufacturer, is not guaranteed or endorsed by the publisher.
